# Perioperative psycho-oncological intervention is associated with reduced psychological distress in women with breast and gynecological cancers: a prospective study

**DOI:** 10.25122/jml-2026-0061

**Published:** 2026-04

**Authors:** Mădălina Daniela Meoded, Mariana Tănase, Claudia Mehedințu, Ciprian Cirimbei

**Affiliations:** 1 Carol Davila University of Medicine and Pharmacy, Bucharest, Romania; 2 Outpatient Psychology Department, Bucharest Institute of Oncology Prof. Dr. Al. Trestioreanu, Bucharest, Romania; 3 Faculty of Psychology and Educational Sciences, Hyperion University, Bucharest, Romania; 4 Department of Obstetrics and Gynecology, Carol Davila University of Medicine and Pharmacy, Bucharest, Romania; 5 Filantropia Clinical Hospital, Bucharest, Romania; 6 Department of Surgery, Carol Davila University of Medicine and Pharmacy, Bucharest Institute of Oncology Prof. Dr. Al. Trestioreanu, Bucharest, Romania

**Keywords:** psycho-oncology, psychological distress, breast cancer, gynecological cancer, attachment, perioperative care, psychological intervention

## Abstract

Psychological distress is highly prevalent among patients with cancer, particularly during the perioperative period. Psycho-oncological interventions may improve emotional outcomes, yet their short-term effectiveness and moderating factors remain insufficiently explored. This prospective pre–post observational study without a control group included 95 women diagnosed with breast and gynecological cancers. Psychological assessments were conducted at baseline (T0) and after a perioperative psycho-oncological intervention (T1), consisting of 3–6 sessions integrating cognitive-behavioral therapy, mindfulness, narrative therapy, and guided imagery. Outcomes included anxiety (HAM-A), depression (HAM-D), post-traumatic stress symptoms (PCL), and self-esteem (RSES). Effect sizes (Cohen’s d) and subgroup analyses were performed. Baseline scores indicated moderate to high levels of psychological distress. Significant improvements were observed across all psychological variables: anxiety (Δ = −7.6, d = 1.10), depression (Δ = −6.1, d = 0.98), PTSD symptoms (Δ = −8.8, d = 1.08), and self-esteem (Δ = +3.7, d = 0.85) (all *P* < 0.001). Clinical response rates ranged from 61% to 68%. Patients with insecure attachment showed higher baseline distress but greater improvement. Educational level, disease stage, and rural vs. urban origin influenced both baseline distress and treatment response. Short-term perioperative psycho-oncological intervention is associated with clinically significant reductions in psychological distress. Response variability suggests the need for personalized approaches based on psychological and socio-demographic profiles.

## Introduction

Psychological distress is highly prevalent among patients with cancer and represents a major challenge in oncological care. Anxiety, depressive symptoms, and trauma-related responses are frequently observed, particularly during critical phases such as diagnosis, hospitalization, and surgical treatment [1,2]. The perioperative period is especially associated with heightened emotional vulnerability, uncertainty, and perceived threat, which may negatively impact both psychological well-being and treatment adherence [3].

In recent years, psycho-oncology has emerged as an essential component of comprehensive cancer care, emphasizing the integration of psychological support into standard medical treatment. Evidence suggests that structured psychological interventions can reduce distress, improve coping mechanisms, and enhance quality of life in patients with cancer [4,5]. However, most studies have focused on long-term interventions or advanced disease stages, while the short-term impact of psycho-oncological support during the perioperative period remains less clearly defined.

Women diagnosed with breast and gynecological cancers represent a particularly vulnerable group. In addition to the physical burden of cancer and its treatment, these patients often experience fear of death, altered body image, and concerns related to femininity and sexuality [6]. These dimensions may further amplify psychological distress and influence the response to therapeutic interventions.

Beyond general distress levels, individual psychological profiles may play a significant role in shaping both baseline emotional vulnerability and response to intervention. Factors such as attachment style, cumulative exposure to stressful or traumatic life events, and socio-demographic characteristics (including educational level and living environment) have been associated with variability in psychological outcomes among patients with cancer [7,8]. Understanding these moderating factors is essential for the development of personalized psycho-oncological approaches.

Despite growing interest in this field, there remains a need for clinically oriented studies that evaluate the effectiveness of short-term, perioperative psycho-oncological interventions using validated psychological measures, while also exploring potential predictors of therapeutic response.

The present study aimed to assess the impact of a structured perioperative psycho-oncological intervention on psychological distress in women with breast and gynecological cancers. Additionally, the study explored the influence of psychological and socio-demographic factors on baseline distress and treatment response, with the goal of contributing to a more individualized approach to psycho-oncological care.

## Material and Methods

### Study design and participants

This prospective pre–post observational study was conducted in a clinical oncology setting and included women diagnosed with breast and gynecological cancers who were undergoing surgical treatments as part of multimodal oncological care. Given the exploratory and clinically oriented nature of the study, no parallel control group was included. A total of 95 patients were consecutively recruited between January 2025 and February 2026 from Bucharest Institute of Oncology “Prof. Dr. Al. Trestioreanu”.

Inclusion criteria were confirmed diagnosis of breast or gynecological malignancy, indication for surgical treatment, age ≥18 years, and ability to provide informed consent and complete psychological assessments.

Exclusion criteria included severe cognitive impairment, active psychotic disorders, or inability to participate in psychological intervention sessions.

All participants provided written informed consent prior to inclusion in the study.

### Psycho-oncological intervention

Participants received a structured perioperative psycho-oncological intervention consisting of 3 to 6 individual sessions delivered by a trained psycho-oncology specialist. The intervention was conducted during the perioperative period (preoperative and early postoperative phases).

Although the intervention was individualized according to patient needs and emotional profile, the overall structure generally included: (1) emotional assessment and psychoeducation regarding perioperative distress; (2) cognitive-behavioral techniques focused on anxiety regulation and maladaptive thoughts; (3) mindfulness and grounding exercises for emotional stabilization; (4) narrative exploration of illness-related fears, traumatic experiences, and meaning reconstruction; and (5) guided imagery and coping-oriented strategies aimed at enhancing emotional adaptation and perceived control. Particular emphasis was placed on narrative therapy elements and the restoration of psychological meaning, given the existential vulnerability frequently associated with cancer diagnosis and surgical treatment.

The number of sessions was adapted to individual patient needs, with an average duration of approximately 45–60 minutes per session.

### Psychological assessment

Psychological assessments were performed at two time points:

- T0 (baseline): prior to initiation of the psycho-oncological intervention (typically preoperatively)
- T1 (post-intervention): following completion of the intervention (approximately 6–8 weeks after baseline)Validated psychometric instruments were used:
- Validated psychometric instruments were used:
- Hamilton Anxiety Rating Scale (HAM-A): to assess anxiety severity
- Hamilton Depression Rating Scale (HAM-D): to evaluate depressive symptoms
- Post-Traumatic Stress Disorder Checklist (PCL): to measure trauma-related distress
- Rosenberg Self-Esteem Scale (RSES): to assess global self-esteem

In addition, attachment patterns were evaluated using the Adult Attachment Scale (AAS), allowing classification into secure, anxious, and avoidant attachment styles, as well as dimensional analysis based on subscales (anxiety, avoidance, closeness).

Socio-demographic data (age, educational level, area of residence) and clinical variables (type of cancer, disease stage) were also collected. All assessments were administered by trained personnel. No patients were lost to follow-up during the study period.

### Outcome measures

The primary outcome was the change in psychological distress between T0 and T1, assessed through differences (Δ) in HAM-A, HAM-D, and PCL scores.

Secondary outcomes included:

- change in self-esteem (RSES)
- proportion of clinical responders (defined as ≥30% reduction in symptom scores)
- associations between psychological/socio-demographic variables and treatment response

### Statistical analysis

Data analysis was performed using SPSS Statistics version 26.0. Continuous variables were expressed as mean ± standard deviation (SD), while categorical variables were presented as frequencies and percentages.

Normality of distribution was assessed using the Shapiro–Wilk test. Pre–post comparisons (T0 vs. T1) were performed using paired *t*-tests for normally distributed variables or non-parametric equivalents where appropriate.

Effect sizes were calculated using Cohen’s d, interpreted as small (0.2), medium (0.5), and large (≥0.8). A significance threshold of *P* < 0.05 was considered statistically significant.

Exploratory subgroup analyses were conducted to evaluate differences according to attachment style, educational level, area of residence (urban vs. rural), and disease stage. Subgroup analyses were exploratory and hypothesis-generating; therefore, no formal correction for multiple comparisons was applied, and findings should be interpreted cautiously.

## Results

### Baseline characteristics

A total of 95 women diagnosed with breast and gynecological cancers were included in the study. The cohort comprised patients with breast cancer (*n* = 40), cervical cancer (*n* = 34), and uterine corpus cancer (*n* = 21), reflecting a heterogeneous oncological population representative of routine clinical practice.

Participants presented a diverse socio-demographic profile, with variability in educational level and area of residence. A notable proportion of patients originated from rural areas and had lower or medium levels of education, factors that were later explored in relation to psychological outcomes.

Attachment assessment revealed a predominance of insecure attachment patterns within the cohort. Anxious attachment was identified in 37.8% of participants, avoidant attachment in 34.5%, while only 27.7% presented a secure attachment style. This distribution suggests a high prevalence of psychological vulnerability factors in this population.

At baseline (T0), patients exhibited moderate to high levels of psychological distress across all domains. Mean scores indicated clinically relevant levels of anxiety, depressive symptoms, and trauma-related distress, supporting the need for targeted psychological intervention in the perioperative setting.

### Changes in psychological outcomes following intervention

Following the perioperative psycho-oncological intervention, statistically significant improvements were observed across all psychological variables assessed, as shown in Table 1.

**Table 1:** Comparison of pre- and post-intervention scores

Parameter	T0	T1	Δ	*P*	d
HAM-A (Anxiety)	21.8	14.2	-7.6	<0.001	1.10
HAM-D (Depression)	19.6	13.5	-6.1	<0.001	0.98
PCL (PTSD symptoms)	36.2	27.4	-8.8	<0.001	1.08
RSES	17.4	21.1	+3.7	<0.001	0.85

Anxiety levels, measured using HAM-A, showed a mean reduction of 7.6 points (*P* < 0.001), corresponding to a large effect size (Cohen’s d = 1.10). This magnitude of change indicates not only statistical significance but also strong clinical relevance.

Depressive symptoms, assessed by HAM-D, decreased by a mean of 6.1 points (*P* < 0.001), with a large effect size (d = 0.98), suggesting substantial emotional improvement during the perioperative period.

Post-traumatic stress symptoms (PCL) demonstrated a mean reduction of 8.8 points (*P* < 0.001), with a large effect size (d = 1.08), highlighting the intervention’s impact on trauma-related distress.

In contrast to reductions in negative psychological symptoms, self-esteem (RSES) significantly increased by 3.7 points (*P* < 0.001), with a large effect size (d = 0.85), indicating a positive shift in patients’ psychological resources.

These changes exceeded commonly accepted thresholds for clinically meaningful improvement. Taken together, these findings suggest a consistent pattern of improvement across both distress-related and adaptive psychological dimensions.

### Clinical response rates

Clinical response, defined as a ≥30% reduction in symptom scores, was observed in a substantial proportion of patients.

Response rates were highest for anxiety (68%), followed by depression (64%) and PTSD-related symptoms (61%). These findings indicate that the majority of patients experienced clinically meaningful psychological improvement from the intervention within a relatively short timeframe.

Importantly, the presence of both statistically significant changes and high responder rates strengthens the clinical relevance of the intervention, suggesting that improvements were not limited to marginal score variations but reflected substantial symptom reduction.

**Figure 1:**
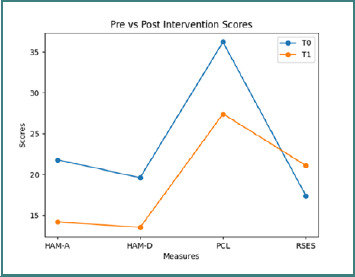
Pre-post evolution of anxiety (HAM-A), depression (HAM-D), PTSD-related symptoms (PCL), and self-esteem (RSES) following perioperative psycho-oncological intervention

A different gradient of response was observed at each level, with the highest response for anxiety, intermediate response for depression, and the lowest response for PTSD, illustrated in Figure 1. We can appreciate that anxiety was the most reactive and rapidly modifiable, while PTSD was more deeply rooted and requires repeated and long-term interventions.

### Exploratory subgroup analyses

#### Attachment style and psychological distress

Patients with insecure attachment styles (anxious and avoidant) exhibited higher baseline levels of psychological distress across all measures compared to those with secure attachment.

Despite their higher initial distress, these patients demonstrated greater absolute reductions in symptom scores following intervention. This pattern suggests that individuals with higher psychological vulnerability may also have greater potential for improvement when appropriate psycho-oncological support is provided.

**Figure 2:**
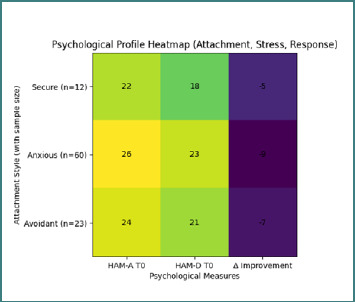
Integrative psychological profile illustrating the relationship between attachment style, baseline distress, and response to psycho-oncological intervention. Values reflect comparative representations derived from observed psychological patterns and were normalized to a 0–10 scale for graphical visualization.

Figure 2 illustrates the relationship between attachment style, baseline psychological distress, and response to psycho-oncological intervention. Patients with insecure attachment patterns (anxious and avoidant) presented higher levels of baseline distress compared to those with secure attachment. At the same time, a differentiated response pattern can be observed: patients with anxious attachment tended to show greater reductions in psychological symptoms, suggesting increased emotional engagement and responsiveness to intervention, whereas patients with avoidant attachment demonstrated more limited improvement, potentially reflecting reduced emotional accessibility and engagement in the therapeutic process. These findings support the role of attachment-related psychological profiles as relevant moderators of both vulnerability and therapeutic response in the psycho-oncological context.

#### Socio-demographic factors

Educational level and area of residence were associated with differences in both baseline distress and treatment response. Patients with lower educational levels presented higher baseline levels of anxiety, depression, and trauma-related symptoms. However, these patients also showed greater reductions in psychological distress following intervention, indicating a potentially higher responsiveness to structured psychological support.

Similarly, patients from rural areas exhibited higher initial distress than those from urban areas. A tendency toward greater improvement was observed in this subgroup, although variability remained.

#### Disease stage

Patients with more advanced disease stages tended to report higher baseline psychological distress (Table 2). However, the relative reduction in symptoms following intervention was comparable across disease stages, suggesting that the benefits of psycho-oncological intervention are not limited by disease severity.

**Table 2:** Distress and evolution according to disease stage

Stage	HAM-A T0	HAM-D T0	PCL T0	Δ HAM-A	Δ HAM-D	Δ PCL
Stage I	18.2	16.5	30.1	-6.1	-5.2	-7.0
Stage II	22.4	20.3	36.8	-7.8	-6.4	-9.2
Stage III + IV	25.9	23.1	41.5	-6.9	-5.8	-7.9

#### Subgroups with poor response: clinical implications

The identification of patients with limited response to intervention is one of the study’s important contributions. These patients were characterized by severe initial distress, complex trauma history associated with avoidant or disorganized attachment styles, and reduced social support.

These findings suggest that standard, short-term interventions may be insufficient for these cases, requiring longer-term interventions, trauma-focused approaches, and ongoing psychological support.

Individual analysis highlighted the existence of a subgroup of patients with limited therapeutic response (non-responders or partial responders), as shown in Table 3. The common characteristics identified were represented by:

- very high initial distress with high scores HAM-A > 28, HAM-D > 25, PCL > 40
- history of severe/complex trauma: abandonment, abuse, major losses
- predominantly avoidant or disorganized attachment style, with difficulties in engaging in the therapeutic process and a tendency to avoid emotions
- poor social context with reduced social support, active relational conflict
- medical factors with advanced stages and complications or reserved prognosis

The differences are marked and confirm the existence of distinct response profiles, with non-responders requiring longer interventions and deeper approaches (e.g., complex trauma).

**Figure 3:**
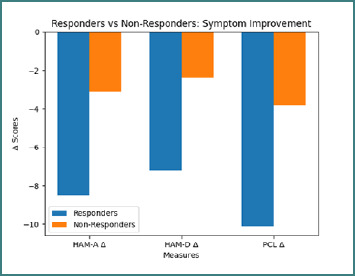
Comparative evolution of psychological symptom reduction (Δ scores) in responders versus non-responders following perioperative psycho-oncological intervention.

Figure 3 highlights significant differences in the magnitude of improvement in psychological symptoms between responders and non-responders. Responders showed marked reductions in anxiety, depression, and posttraumatic stress scores, while non-responders had limited improvements, suggesting the existence of distinct response profiles to psycho-oncological intervention.

These subgroup findings should be considered exploratory and interpreted cautiously, particularly in the absence of correction for multiple comparisons.

**Table 3:** Comparison of regression scores for responders vs. non-responders

Group	Δ HAM-A	Δ HAM-D	Δ PCL
Responders	-8.5	-7.2	-10.1
Non-responders	-3.1	-2.4	-3.8

#### Integrated interpretation of results

Overall, the perioperative psycho-oncological intervention was associated with significant and clinically meaningful reductions in anxiety, depression, and trauma-related symptoms, alongside improvements in self-esteem.

The findings also highlight the role of psychological and socio-demographic factors in shaping both baseline vulnerability and response to intervention. In particular, patients with higher initial distress—such as those with insecure attachment or lower socio-demographic resources—appeared to experience greater psychological improvement following intervention.

## Discussion

The present study showed that a short-term perioperative psycho-oncological intervention was associated with significant reductions in anxiety, depressive symptoms, and trauma-related distress, together with an increase in self-esteem, in women with breast and gynecological cancers. These findings are consistent with the growing body of evidence supporting the role of psycho-oncology as an essential component of comprehensive cancer care [9,10].

Psychological distress in oncology is a multidimensional construct, encompassing anxiety, depression, and trauma-related symptoms, which frequently coexist and interact [11,12]. The consistent improvement observed across all measured variables suggests a broad psychological benefit associated with the intervention.

This finding aligns with previous meta-analyses demonstrating that structured psycho-oncological interventions can produce significant reductions in emotional distress and improvements in quality of life [13,14].

Importantly, the present study extends existing literature by focusing on the perioperative period, a phase characterized by heightened emotional vulnerability, uncertainty, and increased perceived threat [3,15]. While most prior research has focused on survivorship or long-term interventions, our results suggest that even a short-term intervention (3–6 sessions) may yield clinically meaningful benefits when delivered at a critical moment in the disease trajectory.

The magnitude of the observed effects, reflected in large effect sizes across all variables, is notable and comparable to, or even higher than, those reported in previous studies of psychosocial interventions in oncology [16,17]. This suggests that timing may play a key role in intervention effectiveness, with perioperative distress representing a window of heightened psychological accessibility.

A central contribution of this study is the analysis of clinical response rates, which reached 68% for anxiety, 64% for depression, and 61% for trauma-related symptoms. These findings indicate that the majority of patients experienced meaningful symptom reduction, supporting the clinical relevance of the intervention. The differential response across domains is also noteworthy. Anxiety symptoms, often more reactive and situational, showed the highest response rate, while trauma-related symptoms—more closely linked to prior experiences and deeper psychological processes—showed slightly lower responsiveness, consistent with previous literature on trauma and cancer [18,19].

These findings are consistent with prior evidence suggesting that reductions in anxiety and depression are closely associated with improvements in overall quality of life in oncology patients.

Beyond overall symptom reduction, variability in therapeutic response also emerged as a clinically relevant finding.

The distinction between responders and non-responders represents an important and relatively underexplored dimension in psycho-oncological research. Our findings suggest that response to intervention is heterogeneous and influenced by underlying psychological profiles. Non-responders appeared to represent a subgroup with greater psychological complexity, including unresolved trauma, rigid defensive mechanisms, and impaired emotional processing. This interpretation is consistent with models of complex trauma and stress-related psychopathology, which emphasize the role of cumulative stress exposure and impaired emotional regulation [20-22].

Patients classified as responders showed markedly greater reductions in anxiety, depressive, and trauma-related symptoms compared to non-responders, with differences exceeding twofold across all measures. This pattern indicates that the intervention effect was not uniform across the cohort but instead revealed distinct response profiles. The presence of a subgroup with limited improvement further underscores the complexity of psychological adaptation in oncology and suggests that, while short-term interventions are effective for most patients, a subset may require more intensive or longer-term, tailored psycho-oncological support.

The attachment theory provides a useful framework for understanding these differences. In our study, patients with insecure attachment patterns exhibited higher baseline distress, in line with previous findings linking attachment insecurity to increased vulnerability in medical contexts [23]. At the same time, some of these patients demonstrated substantial improvement following intervention, suggesting that higher baseline distress may also reflect increased emotional accessibility and responsiveness to therapeutic engagement. Conversely, avoidant attachment patterns may be associated with reduced emotional expression and lower engagement, potentially limiting short-term intervention effects [24].

Socio-demographic factors also played a relevant role. Patients with lower educational levels and those from rural environments exhibited higher baseline distress, consistent with literature highlighting the impact of social determinants on psychological outcomes in cancer [21-23]. These disparities may reflect differences in access to information, coping resources, and psychosocial support. Notably, these patients also showed meaningful improvements following intervention, suggesting that psycho-oncological care may help reduce inequalities in emotional burden.

With respect to disease stage, our findings confirm that advanced disease is associated with higher psychological distress [24]. However, the benefits of intervention were observed across stages, supporting the integration of psycho-oncological care throughout the disease trajectory. This aligns with current recommendations emphasizing the need for systematic distress screening and psychosocial support in oncology [2,25].

An additional important aspect of this study is the indirect evidence of quality-of-life improvement. Although quality of life was not assessed using a dedicated instrument such as the EORTC QLQ-C30, the observed reductions in anxiety, depression, and trauma-related symptoms, together with increased self-esteem, strongly suggest an overall improvement in patients’ psychological well-being. Previous research has shown that these dimensions are closely linked to quality-of-life outcomes in oncology [5,10,11].

Beyond symptom reduction, the observed responder rates and increase in self-esteem suggest broader improvements in emotional adjustment and subjective well-being, dimensions closely related to quality of life in oncology populations.

From a clinical perspective, the increase in self-esteem is particularly meaningful, as it may reflect not only symptom reduction but also a process of psychological adaptation and restoration of personal agency. In the context of cancer, where identity and body image may be profoundly affected, improvements in self-esteem may represent a key mechanism underlying broader quality-of-life changes [6].

Beyond the psychological dimension, the observed improvements may also be interpreted in light of emerging evidence from psycho-neuro-immunology. Chronic stress and unresolved psychological distress have been associated with dysregulation of the hypothalamic–pituitary–adrenal (HPA) axis, increased cortisol secretion, and alterations in immune function, including reduced natural killer cell activity and increased pro-inflammatory cytokine levels [16,26,27]. Previous studies have suggested potential associations between psychological interventions and neuroendocrine or immune regulation [27,28]. In this context, the reductions in anxiety, depression, and trauma-related distress observed in our study may be conceptually consistent with previously described psycho-neuro-immunological mechanisms. However, these biological pathways were not directly assessed in the present study.

In addition, growing evidence suggests that perioperative psychological state may influence clinical outcomes, including recovery, complication rates, and treatment adherence. Elevated preoperative anxiety has been associated with increased postoperative pain, longer hospital stays, and poorer functional recovery [3,29]. Conversely, psychological interventions aimed at reducing distress have been shown to improve perioperative adaptation and, in some cases, treatment compliance [29,30]. Although our study did not assess surgical or oncological outcomes directly, the substantial reduction in psychological distress and the high proportion of responders suggest that perioperative psycho-oncological support may have broader implications beyond emotional well-being, potentially contributing to improved overall care trajectories.

An important contribution of the present study lies in several elements of novelty that extend the current psycho-oncological literature. First, the study specifically focuses on the perioperative period, a critical yet relatively underexplored phase in which psychological vulnerability is heightened, but intervention opportunities are often limited. Second, it evaluates the effectiveness of a short-term, structured psycho-oncological intervention (3–6 sessions) and suggests that clinically meaningful improvements may be achievable within a limited timeframe, which is highly relevant to real-world clinical settings.

Third, the study goes beyond traditional pre–post comparisons by incorporating clinical response rates, providing a more practice-oriented perspective on treatment effectiveness. Fourth, the explicit differentiation between responders and non-responders offers a more nuanced understanding of therapeutic outcomes and highlights the heterogeneity of psychological adaptation in oncology patients. This aspect remains relatively underexplored in existing psycho-oncological research.

Fifth, the integration of attachment-related psychological profiles as potential moderators of both baseline distress and treatment response contributes to a more personalized conceptual framework, linking relational patterns with clinical outcomes. Finally, although not assessed using a dedicated instrument, the study provides indirect evidence of quality-of-life improvement by demonstrating concurrent reductions in distress and increases in self-esteem, suggesting broader implications for patient well-being.

Taken together, these findings support a personalized model of psycho-oncological care, in which intervention strategies are adapted to individual psychological profiles, socio-demographic context, and clinical characteristics. The identification of responders and non-responders may be particularly useful in guiding stepped-care approaches and optimizing resource allocation.

### Limitations

Several limitations should be acknowledged. First, the absence of a control group limits the ability to establish causal relationships between the intervention and the observed improvements. Although the pre–post design provides valuable insight into changes over time, the observed effects may also be influenced by factors such as natural psychological adaptation, perioperative medical care, or regression to the mean.

Second, the observational design does not control for spontaneous adaptation or treatment-related effects. Third, subgroup analyses were exploratory and should be interpreted with caution. Fourth, quality of life was inferred indirectly rather than assessed using a standardized instrument.

Finally, the relatively short follow-up period does not allow conclusions regarding the long-term sustainability of the observed effects.

In addition, the lack of randomization may introduce selection bias, limiting the generalizability of the findings. Potential therapist-related effects and variability in the number of intervention sessions (3–6 sessions) may also have influenced outcomes and should be considered when interpreting the results.

### Clinical implications and future directions

Despite these limitations, the study has practical implications. It suggests that psycho-oncological intervention delivered during the perioperative period may be both feasible and clinically relevant, even when brief. It also underscores the importance of identifying psychological profiles associated with differential responses to optimize care pathways. Future studies should include controlled designs, longer follow-up, and dedicated quality-of-life instruments, while further exploring the role of attachment, trauma history, and social context as moderators of psycho-oncological outcomes.

## Conclusion

This prospective study suggests that a structured, short-term psycho-oncological intervention delivered during the perioperative period is associated with significant and clinically meaningful improvements in psychological distress among women with breast and gynecological cancers.

The findings consistently showed heterogeneous response patterns influenced by psychological and socio-demographic factors, particularly attachment-related vulnerability. The distinction between responders and non-responders, together with the observed improvements in distress-related domains and self-esteem, supports the clinical relevance of integrating more individualized psycho-oncological strategies into standard oncological care.

Taken together, these findings support the integration of perioperative psycho-oncological interventions as a clinically meaningful component of cancer care, with the potential to improve psychological well-being, enhance quality of life, and contribute to more personalized, patient-centered therapeutic strategies.
